# Has India’s national rural health mission reduced inequities in maternal health services? A pre-post repeated cross-sectional study

**DOI:** 10.1093/heapol/czw100

**Published:** 2016-08-10

**Authors:** Sukumar Vellakkal, Adyya Gupta, Zaky Khan, David Stuckler, Aaron Reeves, Shah Ebrahim, Ann Bowling, Pat Doyle

**Affiliations:** 1 Center for Chronic Diseases and Injuries, Public Health Foundation of India, Gurgaon, Haryana, Postal code 122002, India; 2 Department of Sociology, University of Oxford, Oxford, OX1 3UQ, United Kingdom; 3 Department of Non-communicable Disease Epidemiology, London School of Hygiene and Tropical Medicine 15-17 Tavistock place, London, WC1H 9SH, United Kingdom; 4 Department of Health Sciences, University of Southampton, Southampton, SO17 1BJ, United Kingdom

**Keywords:** Antenatal care, institutional delivery, Indian states, maternal healthcare, National Rural Health Mission, public health program, socioeconomic inequity

## Abstract

**Background:** In 2005, India launched the National Rural Health Mission (NRHM) to strengthen the primary healthcare system. NRHM also aims to encourage pregnant women, particularly of low socioeconomic backgrounds, to use institutional maternal healthcare. We evaluated the impacts of NRHM on socioeconomic inequities in the uptake of institutional delivery and antenatal care (ANC) across high-focus (deprived) Indian states.

**Methods:** Data from District Level Household and Facility Surveys (DLHS) Rounds 1 (1995–99) and 2 (2000–04) from the pre-NRHM period, and Round 3 (2007–08), Round 4 and Annual Health Survey (2011–12) from post-NRHM period were used. Wealth-related and education-related relative indexes of inequality, and pre-post difference-in-differences models for wealth and education tertiles, adjusted for maternal age, rural-urban, caste, parity and state-level fixed effects, were estimated.

**Results:** Inequities in institutional delivery declined between pre-NRHM Period 1 (1995–99) and pre-NRHM Period 2 (2000–04), but thereafter demonstrated steeper decline in post-NRHM periods. Uptake of institutional delivery increased among all socioeconomic groups, with (1) greater effects among the lowest and middle wealth and education tertiles than highest tertile, and (2) larger equity impacts in the late post-NRHM period 2011–12 than in the early post-NRHM period 2007–08. No positive impact on the uptake of ANC was found in the early post-NRHM period 2007–08; however, there was considerable increase in the uptake of, and decline in inequity, in uptake of ANC in most states in the late post-NRHM period 2011–12.

**Conclusion:** In high-focus states, NRHM resulted in increased uptake of maternal healthcare, and decline in its socioeconomic inequity. Our study suggests that public health programs in developing country settings will have larger equity impacts after its almost full implementation and widest outreach. Targeting deprived populations and designing public health programs by linking maternal and child healthcare components are critical for universal access to healthcare.

Key Messages

- India’s National Rural Health Mission (NRHM), one of the largest public health programs in the world launched in 2005, increased the uptake of institutional delivery and antenatal services, particularly among the poorest socioeconomic groups, in the less-developed high-focus Indian states.
- Although the uptake of antenatal services were not improved in the early post-NRHM period 2007–08, there was considerable increase in the uptake of, and decline in its inequity in the late post-NRHM period 2011–12.
- Larger equity impacts in the uptake of institutional delivery and antenatal services were found in the late post-NRHM period 2011–12 than in the early post-NRHM period 2007–08, indicating that public health programs in developing country settings will have larger equity impacts after its almost full implementation and widest outreach.
- Impact of NRHM was greatest in those states with higher proportion of beneficiaries enrolled under conditional cash-transfer program of Janani Suraksha Yojana.
- Targeting deprived populations and designing public health programs by linking maternal and child healthcare components are critical for universal access to healthcare.

## Introduction

India has the highest number of maternal and infant deaths worldwide and accounts for one-fifth of all global maternal mortalities, and 21% of the children of less than five dying every day in the world are Indians (International Institute for Population Sciences and Macro International 2007; The World Bank 2014). There exist large inequalities in maternal and infant mortality rates across Indian states, as well as significant gaps between wealthy and deprived groups within these states (International Institute for Population Sciences and Macro International 2007; International Institute for Population Sciences and Macro International 2010). Children from the poorest communities are more likely to die before they reach the age of 5 (Save the Children 2010) and stillbirths and neonatal mortality rates are higher than those of higher income groups (Joshi 2009). Promoting maternal and child health services such as ante-natal care (ANC), institutional delivery and child immunisation reduces maternal and infant mortality rates (Martines *et al.* 2005; World Health Organisation 2005; Langlois 2013). Even though most of the primary healthcare in public health facilities is available free of charge, the use of maternal and child health services are still relatively low with considerable socioeconomic inequity within and across the Indian states (Pallikadavath *et al.* 2004; Vora *et al.* 2009; Pathak *et al.* 2010; Sanneving *et al.* 2013; Joe 2014).

To address these longstanding inequalities, the Indian government launched National Rural Health Mission (NRHM) in 2005. Its aim was to strengthen the primary healthcare system. One of the key thrusts here was to encourage pregnant women, particularly those of low socioeconomic backgrounds, to use institutional maternal and child healthcare. The NRHM had a set of core strategies including increasing public health funding, decentralising village and district level health planning and management, strengthening the public health service delivery infrastructure, particularly at village, primary and secondary levels, and promoting the non-profit sectors to increase social participation and community empowerment (Planning Commission of India 2001; Government of India 2014b; Sharma and Joe 2014). To ensure wide outreach the NRHM employed ‘Accredited Social Health Activists’ (ASHA) at the grass-roots (village) level to support the use of services (Government of India 2014). One of the important components of the NHRM was the ‘Janani Suraksha Yojana’ (JSY), a cash-transfer programme, which provided financial support to enable women from lower socio-economic groups to give birth in a health facility (Lim *et al.* 2010; Government of India 2014a).

NRHM implementation varied across the 18 high-focus (deprived) and the 10 low-focus (developed) states, determined by maternal and child health indicators. On one hand, the high-focus states, where the program was first rolled out, were entitled to more funds from the central government and additional technical and managerial support. Furthermore, in the high-focus states, all pregnant women were eligible for JSY financial support of Indian rupee (INR) 1400 (∼ US$25) per birth, and benefits were paid irrespective of the birth order, age and socioeconomic position. On the other hand, the JSY financial assistance of INR 800 (∼ US$14) in the low-focus states was limited to women who are below the poverty line, married and aged 19 or more.

Research has found that NRHM’s JSY payments were associated with increases in health facility births and decline in neonatal mortality (Lim *et al.* 2010; Gupta *et al.* 2012; Panja *et al.* 2012; Randive*et al.* 2013), improvement in immunization rates and breastfeeding practices (Carvalho 2014), decline in economic inequality in institutional delivery in the districts of higher JSY coverage, and decline in maternal mortality in richest districts than in the poorest (Randive 2014). Most of the available studies examined the effects specific to JSY payments, by defining those who had received JSY financial payment as treatment groups, and those who did not as control groups. Understanding the population level impacts of NRHM are critical for India’s health policy and planning, particularly when the country is thriving to achieve its aspirations to attain universal healthcare coverage through exploring several alternative strategies including supply-side strengthening and demand-side financing. To our knowledge, studies have yet to assess the population-level impact of NRHM. Furthermore, no studies have examined the impacts of the program design of NRHM. JSY is one of the major components of NRHM focusing on promoting the uptake of institutional delivery through conditional cash-transfer to mothers and ASHAs. This cash-transfer to mothers was not linked to the uptake of antenatal care (ANC). Any impact of NRHM might be driven by the JSY’s conditional cash-transfer for the uptake of institutional delivery, and so may neglect other components of primary healthcare such as ANC, at least in the early post-NRHM period. However, from 2009 to 2010 onwards, several state governments revised the JSY guidelines to also promote the provision of ANC. For instance, in 2009, the Chhattisgarh government made ensuring the provision of ANC one of the eligibility conditions for payment of incentives to ASHAs (Government of Chhattisgarh 2009). Furthermore, the NRHM framework of implementation suggests that the full implementation of the program with wider outreach will be attained over time (Government of India 2016). For example, the NRHM aimed to achieve 50% coverage of the villages with fully trained ASHA by 2007, and 100% by 2008. Nonetheless, the target of setting up of Village Health and Sanitation Committee in each village was 30% by 2007 and 100% by 2008, and the target of setting up (and strengthening) of Sub Health Centres with two auxiliary nurse midwives (ANM) employed was 60% by 2009 and 100% by 2010. The aim was for most targets to be met by the end of 2010, while the remainder by the end of 2012. However, most of the available studies that assessed the effects of JSY were based on the early post-NRHM period data of 2006–08, thus excluding the program effects after almost full implementation and widest outreach.

Here, using a difference-in-differences study design, we evaluated the impact of NRHM on socioeconomic inequity in the uptake of institutional delivery and ANC. Since the JSY of NRHM gives cash-transfers to mothers and ASHAs conditional on using institutional delivery, whereas no cash-transfer is supplied for the use of ANC until 2010 to the ASHAs, we hypothesise that the impact of NRHM is more likely to be seen in increased uptake of, and reduced socioeconomic inequity in institutional delivery, but not on ANC use in the early post-NRHM period. Based on the data availability, we classified the post-NRHM period into ‘early post-NRHM-period 2007–08’ and ‘late post-NRHM period 2011–12’. We further hypothesise that greater equity impacts of NRHM in terms of uptake of institutional delivery and ANC is likely to be seen in the late post-NRHM period of widest outreach of the program than in the early post-NRHM period.

## Methods

### Study design

Using a quasi-natural experiment study design, we analysed four national-level cross-sectional survey datasets in the pre- and post NRHM periods.

### Study samples

Of the 18 high-focus states, we considered eight empowered action group (EAG) states and seven north-eastern (NE) states in this analysis. The EAG states, where 46% Indian population live, were lagging behind in containing population growth and, compared with the rest of the Indian states, had poorer socio-economic, demographic and health indicators. Thus, the committee named ‘Empowered Action Group’, set up by the government of India in 2001, recommended to pay particular attention to these eight states in terms of area-specific programs and action plans for efficient service delivery in collaboration with various ministries of the union and state governments. The NE states also were socioeconomically less developed, and geographically isolated from the rest of India, and represents about 4% of the total Indian population. We excluded the state of Nagaland (an NE state) in our analysis because DLHS-3 was not implemented there. We also excluded the high-focus states of Jammu & Kashmir and Himachal Pradesh. These two states are socioeconomically developed with better health outcomes in terms of lower maternal and infant mortality rates along with higher levels of the uptake of institutional health services than the EAG states. Indeed their development is at a similar level to several low-focus NRHM states. For instance, about 68% and 80% of pregnant women had minimum three ANC visits and 45% and 71% had institutional delivery in the pre-NRHM period (2000–04) in Himachal Pradesh and Jammu & Kashmir, respectively.

We used data from the repeated cross-sectional surveys of married women from the District Level Household and Facility Surveys (DLHS) Round 1 in 1995–99, Round 2 in 2000–04, Round 3 in 2007–08, Round 4 in 2011–12 and the Annual Health Survey (AHS) in 2011–12. The DLHS Round 4 was not implemented in the EAG states and Assam (one of the NE state). Instead, the AHS was conducted in these states with relatively larger sample size than the DLHS. The AHS followed the survey methodology and survey instruments consistent with the DLHS. The AHS was conducted by the census office, the Government of India, and the DLHS was conducted by the International Institutes for Population Sciences (IIPS Mumbai), on behalf of the Ministry of Health and Family Welfare, Government of India. The IIPS is an international research institute and is responsible for the design, development of survey tools and software, training of regional agencies entrusted to undertake the fieldwork in different states, quality assurance and the overall supervision and management of DLHS. The DLHS datasets are available for secondary data analysis for research purposes at nominal cost from IIPS, Mumbai, and the AHS data set from the office of the census of India. The details of the survey methods and survey instruments are available in the national level overview reports [http://www.rchiips.org/andhttp://censusindia.gov.in/]. Briefly, the surveys followed a systematic, multi-stage stratified sampling design. Women were included in the study sample if they reported a live birth, stillbirth or spontaneous or induced abortion within a specified period of the interview date. This period was an average of the past 4 years, being 1 January 1995 for DLHS-1, 2000 for DLHS-2, 2004 for DLHS-3 and 2008 for DLHS-4. We included a reduced time window of 2007–08 for DLHS-3 to allow for delays in implementation of NRHM across the country (this resulted in relatively smaller analytical sample size for DLHS-3 compared with DLHS-1 and DLHS-2). In the DLHS rounds, data on the use of maternal healthcare were collected for the most recent birth. In the AHS, however, the collection of data on the uptake of maternal healthcare was limited to the period of 1 January 2011–31 December 2011. To be consistent with the AHS data period, we included the reduced time window of 2011–12 for DLHS-4 (rather than 2008–12). We retained the period 2012 in the DLHS-4 to avoid the loss of sample size.

The respondents of DLHS-1 and 2 were currently married women whereas the respondents of DLHS-3 and DLHS-4/AHS were ever-married women. Thus DLHS-3 and DLHS-4/AHS were more inclusive of mothers that were divorced, single or widowed. The age profile of the respondents varied across the four rounds. DLHS-1 and DLHS-2 included women aged 15–44, but DLHS-3 and DLHS-4/AHS included women 15–49. We, therefore, excluded women who were aged 45–49 to ensure consistency across the survey rounds. Missing observations relating to institutional delivery and ANC were excluded from the analysis. The final analytical sample size of married women aged 15–44 years, who had reported a live birth, stillbirth, or spontaneous or induced abortion within a specified period of the interview date, was 131 531 in DLHS-1, 135 035 in DLHS-2, 65 090 in DLHS-3 and 400 702 in DLHS-4/AHS. To adjust for sample selection and post-stratification factors in the analysis, state-level sampling weights were used. The data analysis was performed with *Stata 13.0* (StataCorp 2014).

### Definition of variables

The outcome variables were the uptake of institutional delivery and ANC, both dichotomous variables (Yes = 1; No = 0). Institutional delivery is defined as delivery in a healthcare facility of any type. For ANC, we adhered to the JSY recommended number of at least three ANC visits, either at a healthcare facility or a healthcare worker visiting pregnant women to give ANC.

The location of residence (rural/urban), age of women at interview, years of highest education (either of the respondent or husband, whichever was the highest) and asset index were included in the analysis. Asset index (a proxy for wealth and income) and years of highest education were used as two distinct measures of socioeconomic position. The asset index used for the DLHS-3 was the one originally provided in the dataset which was estimated using principal component analysis of variables such as ownership of house and its features, including toilet, electricity connection, cooking gas, fridge, fan, television, radio, sewing machine and vehicles. Asset scores for DLHS-1, DLHS-2 and DLHS-4/AHS were generated by the research team using the same approach (Filmer and Pritchett 2001).

### Analytical steps and statistical tools

The analysis followed two major steps. First, using the asset-related and education-related relative index of inequality (RII), we measured the population level socioeconomic inequities in the uptake of institutional delivery and ANC in the pre- and post-NRHM periods. The RII is a regression-based measure of inequalities that takes the whole socioeconomic distribution of population into account. When the RII > 1 relatively more women of higher socioeconomic position than lower socioeconomic position utilise maternal healthcare, and vice-versa for < 1. Although other measures of inequalities are available, such as concentration index and absolute slope index of inequality, the RII is recommended for performing comparisons over time or across populations (Kunst and Mackenbach 1990; Ernstsen *et al.* 2012).

We measured socioeconomic position using asset indices and educational attainments. To estimate the RII, we transformed these into a summary measure, namely a ridit-score (separately for asset and education groups), scaled from zero to one by arranging the groups in order from lowest to highest socioeconomic position and assigning the cumulative proportion of the population to each group and is weighted to reflect the share of the sample at each asset score and educational attainment (Harper and Lynch 2006). We used generalised linear models (log-binomial regression), with a logarithmic link function to calculate RIIs (rate ratios) (Barros and Hirakata 2003; Spiegelman and Hertzmark 2005; Ernstsen*et al.* 2012). Trends in RII over time were assessed by the inclusion of the interaction term of ridit-score and time, and the corresponding *P*-value was reported in the study (Harper and Lynch 2006).

Second, using the pre-post difference-in-differences (DiD) models adjusted for maternal age, parity, rural-urban, caste and state-level fixed effects, we estimated the effects of NRHM for each wealth and education tertile. In addition to the reference period data of pre-NRHM period of 2000–04 (DLHS-2), we also have included the pre-NRHM period data of 1995–99 (DLHS-1) to capture the pre-NRHM trends in institutional delivery and ANC. Various socioeconomic groups had differential uptake of maternal health services in the pre-NRHM period and several confounders (other than NRHM), such as various poverty alleviation programs and economic growth, would differentially affect the socioeconomic tertiles over time. Moreover, the JSY was launched by modifying the existing National Maternity Benefit Scheme (NMBS). The NMBS provided financial assistance of INR 500/- per birth (up to two live births) to pregnant women aged 19 or more belonging to below the poverty line (BPL) households. Thus, the use of two pre-NRHM period data sets would allow us to control for the effect of NMBS on the uptake of maternal healthcare. The DLHS data meet the two key assumption of DiD method, namely the parallel trends assumption and the stable unit treatment value assumptions. Per the parallel trends assumption, the treatment and the control group would follow the same time trend in the absence of the treatment, and any change in data collection should not influence the trends. To our knowledge, there were no specific changes in the data collection over time to influence the trends as the DLHS data used the same sampling framework and variables of interest across the three rounds without any systematic undercounts or over counts for one group. Similarly, the DLHS data satisfy the stable unit treatment value assumption as there were no observable spill-over effects between treated units. We treated the lowest and middle wealth (and education) tertile as the target group, and the highest tertile as the non-target group. The basic form of our DiD model is the following:

$${\text{Y}}_{i}\;=\;\beta_{0}\;+\;\beta_{\text{1}}.{\text{NRHMYear}}_{\text{i}}\;+\;\beta_{\text{2}}.{\text{BaseYear}}_{\text{i}}\;+\;\beta_{\text{3}}.{\text{SEP}}_{\text{it}}\;+\;\beta_{\text{4}.}{\text{NRHMYear}}_{\text{i}}{}^{*}{\text{BaseYear}}_{\text{i}}{}^{*}{\text{SEP}}_{\text{it}}\;+\;\beta_{\text{5}}.{\text{parity}}_{\text{it}}\;+\;\beta_{\text{6}.}{\text{age}}_{\text{it}}\;+\;\beta_{\text{7}.}{\text{location}}_{\text{it}}{}_{\;}+\;\beta_{\text{8}.}{\text{caste}}_{\text{it}}{}_{\;}+\;\beta_{\text{9}.}{\text{state}}_{\text{it}}+\;{\text{e}}_{\text{it}}$$

where *Y_i_* is the outcome indicator (institutional delivery or ANC) for respondent ‘*i*’; NRHMYear =1 if post-NRHM period (either 2007–08 or 2011–12, as we estimated two models separately for each post-NRHM period) and 0 if DLHS-2 (2000–04); BaseYear = 1 if pre-NRHM period of DLHS-2 (2000–04) and 0 if DLHS-1 (1995–99); SEP is defined to three socioeconomic (wealth or education) tertiles; parity = 1 if the total number of reported live birth, stillbirth, or spontaneous or induced abortion is > 1, and 0 if otherwise; age denotes age of the respondent, location = 1 if the respondent live in urban location and 0 if in rural; caste is defined as three categories of caste, namely scheduled caste/tribe, and forward caste; state is defined as each individual state to capture the state-level fixed effects. The coefficients of the triple interaction terms measure the effects of NRHM for each socioeconomic tertile.

## Results

Table 1 describes the four study samples. The mean age of the women was 27.3 years in DLHS-1, 26.4 years in DLHS-2, 25.4 years in DLHS-3 and 26.1 years in DLHS-4/AHS.

**Table 1.** Description of the study samples

	DLHS-1 (1995–99)	DLHS-2 (2000–04)	DLHS-3 (2007–08)	DLHS-4/AHS (2011–12)
*N*	131,531	135,035	65,090	400,702
Age (mean and SD)	27.3[5.7]	26.4[5.7]	25.4[5.3]	26.1[4.9]
Years of education (mean and SD)	2.6[4.1]	3.5[4.7]	3.7[4.5]	4.1[3.8]
Years of highest education in the family (mean and SD)	5.9[5.2]	6.3[5.2]	6.8[4.9]	6.9[4.9]
Rural (%)	85.0	80.1	74.5	73.3
*Caste*				
SC (%)	19.6	21.0	21.4	20.3
ST (%)	10.4	10.7	17.7	10.5
Other caste (%)	69.9	68.1	60.9	69.2
Minimum three ante-natal care visits (%)	25.9	32.0	34.1	58.4
Institutional delivery (%)	20.6	26.6	39.1	65.4
Skilled birth attendance at home (%)	12.2	13.7	5.1	7.7
JSY finance benefit recipients (%)			18.0	49.1

*Source*: Authors estimates from the DLHS and the AHS data.

### Changes in uptake of institutional delivery and ANC

**Figure 1.**
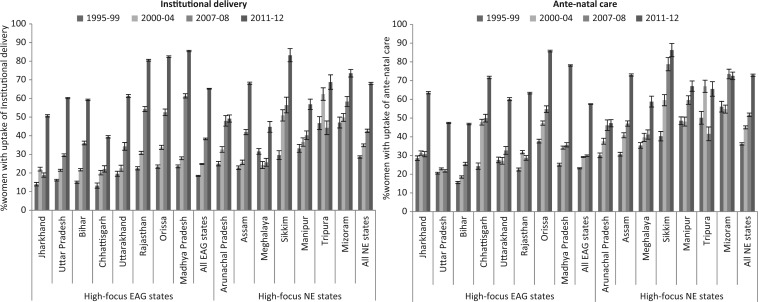
Percentage of eligible women, age 15–44, using institutional delivery and ante-natal care in pre-NRHM periods (1995–99 and 2000–04) and post-NRHM periods (2007–08 and 2011–12), in high-focus empowered action group (EAG) and north eastern (NE) Indian states. *Notes*: (i) Authors estimates from the DLHS and the AHS data; ii) Error bars show 95% confidence intervals.

Most EAG and NE states had experienced considerable increase in the uptake of institutional delivery from pre-NRHM Period 2 to post-NRHM periods as compared with the change in the uptake from pre-NRHM Period 1 to pre-NRHM Period 2 (Figure 1). For instance, in the EAG states as a whole, there was an increase of 13 and 40 percentage points in the uptake of institutional delivery in the early post-NRHM period 2007–08 (38.3%) and late post-NRHM period 2011–12 (65.5%), respectively, from pre-NRHM Period 2 of 2000–04 (24.8%) as against an increase of 7 percentage points in pre-NRHM Period 2 from pre-NRHM Period 1 of 1995–99 (18.5%) (Supplementary Table S1). Similarly, on one hand, in the NE states, there was an increase of 8 and 33 percentage points in the early post-NRHM period 2007–08 (42.6%) and late post-NRHM period 2011–12 (68.0%) as against the increase of 6 percentage points in pre-NRHM Period 2 (34.9%) from pre-NRHM Period 1(28.6%). On the other hand, there was no significant improvement in the uptake of ANC in the EAG states (23.2% in 1995–99, 29.2% in 2000–04 and 29.9% in 2007–08) but a moderate increase was found in the NE states (36.2% in 1995–99, 45.0% in 2000–04 and 51.8% in 2007–08) in the early post-NRHM period 2007–08. However, there was considerable improvement in the uptake of ANC in the late post-NRHM period 2011–12 (57.5% in the EAG and 72.9% in the NE states).

### Trends in inequity of the uptake of institutional delivery and ANC

**Figure 2.**
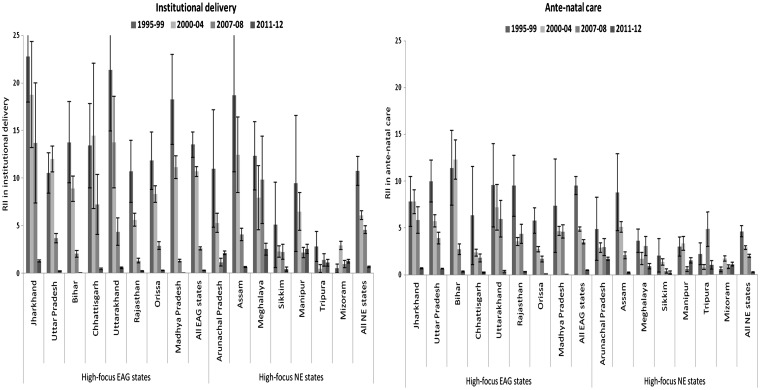
Wealth-related relative index of inequality (RII) in the uptake of institutional delivery and ante-natal care in pre-NRHM periods (1995–99 and 2000–04) and post-NRHM periods (2007–08 and 2011–12), in high-focus empowered action group (EAG) and north eastern (NE) Indian states. *Notes*: (i) Authors estimates from the DLHS and the AHS data. (ii) The RII value reported in the figure is (RII-1); (iii) positive values of the reported RII denotes inequity in favour of the rich; (iv) error bars denote 95% confidence intervals.

Figure 2 and Supplementary Tables S2 and S3 show the estimates of RII. Large socio-economic inequities in the uptake of institutional delivery and ANC, favouring higher socioeconomic groups, were found in the pre-NRHM Periods 1 and 2. A similar pattern was observed in the post-NRHM periods, but the magnitude of inequity in institutional delivery dropped considerably. For example, in the EAG states the wealth-related RII for institutional delivery fell from 14.5 [95% CI: 13.2; 15.9] in 1995–99 to 11.7 [95% CI: 11.2; 12.2] in 2000–04 (*P* of trend < 0.001) to 3.6 [95% CI: 3.5; 3.8] in 2007–08 (*P* of trend < 0.001) to 1.3 [95% CI: 1.3; 1.3] in 2011–12 (*P* of trend < 0.001). Although there was only a moderate decline in inequity in the uptake of ANC between the pre- and the early post-NRHM period 2007–08, there was considerable decline in the late post-NRHM period 2011–12. In the EAG states, the wealth-related RII fell from 9.3 [95% CI: 8.2; 10.6] in 1995–99 to 5.9 [95% CI: 5.6; 6.1] in 2000–04 (*P* of trend < 0.001) to 4.5 [95% CI: 4.3; 4.8] in 2007–08 (*P* of trend < 0.001) to 1.5 [95% CI: 1.5; 1.5] in 2011–12 (*P* of trend < 0.001). Similar pattern was found in the NE states.

### Effects of NRHM

The above-stated changes in inequity in the uptake of institutional delivery were evident for each socioeconomic tertile too, as shown by the observed probability and predicted probability in the uptake for each wealth (Supplementary Figure S1) and education tertile (Supplementary Figure S2). The observed probability in the uptake of institutional delivery was greater than the predicted probability in both EAG and NE states for each socioeconomic tertile, which means a positive impacts of NRHM.

The estimated effects of NRHM showed that each socioeconomic tertile in most of the EAG and NE states had positive program effect in the uptake of institutional delivery in the early post-NRHM period 2007–08 (Table 2). The lowest and middle tertiles had greater impact in the uptake than the highest wealth and education tertiles, particularly in the EAG states. In the EAG states, the net increase in the uptake of institutional delivery in the early post-NRHM period 2007–08 was 13.3% (*β* = 0.133; *P* < 0.001), 15.7% (*β* = 0.157; *P* < 0.001) and 5.3% (*β* = 0.053; *P* < 0.001) for the lowest, middle and highest wealth tertile, respectively. That is, in the EAG states, there was 8.05% (95% CI: 7.98%, 8.12%) and 10.36% (95% CI: 10.28%, 10.44%) higher differential increase in the uptake of institutional delivery for the lowest and middle tertiles over the highest wealth tertile, respectively. In the NE states, however, the uptake of institutional delivery increased, but with a lesser magnitude of 4.4% (*β* = 0.044; *P* < 0.01), 5.9% (*β* = 0.059; *P* < 0.001) and 3.7% (*β* = 0.037; *P* < 0.05) for the lowest, middle and highest wealth tertiles, respectively. That is, there was a moderate differential increased the uptake of 0.7% (95% CI: 0.7%, 0.8%) for the lowest and 2.2% (95% CI: 2.2%, 2.3%) for the middle tertiles over the highest wealth tertile.

**Table 2.** Effects of NRHM in the uptake of institutional delivery and ante-natal care among wealth and education tertiles in the early post-NRHM period 2007–08, in high-focus empowered action group (EAG) and north eastern (NE) Indian states

	Institutional delivery						Ante-natal care					
	Wealth tertiles			Education tertiles			Wealth tertiles			Education tertiles		
	Lowest tertile (1)	Middle tertile (2)	Highest tertile (3)	Lowest tertile (4)	Middle tertile (5)	Highest tertile (6)	Lowest tertile (7)	Middle tertile (8)	Highest tertile (9)	Lowest tertile (10)	Middle tertile (11)	Highest tertile (12)
**EAGstates**												
Jharkhand	−0.02 ±0.02	0.03 ±0.02	0.01 ±0.03	0.02 ±0.01	−0.02 ±0.02	−0.02 ±0.03	0.05 ±0.02**	0.14 ±0.03*	0.11 ±0.03**	0.07 ±0.02**	0.07 ±0.02**	0.03 ±0.03
Uttar Pradesh	0.07 ±0.01*	0.06 ±0.01*	−0.02 ±0.01	0.08 ±0.01*	0.09 ±0.01*	0.07 ±0.01*	−0.06 ±0.01*	−0.09 ±0.01*	−0.15 ±0.01*	0.01 ±0.01	0.02 ±0.01	−0.02 ±0.01
Bihar	0.13 ±0.01*	0.21 ±0.01*	0.09 ±0.02*	0.16 ±0.01*	0.12 ±0.01*	−0.04 ±0.02	0.07 ±0.01*	0.13 ±0.01*	−0.05 ±0.02**	0.15 ±0.01*	0.06 ±0.01*	−0.17 ±0.02*
Chhattisgarh	0.04 ±0.02***	0.07 ±0.03**	−0.07 ±0.04***	0.04 ±0.02***	0.09 ±0.03*	−0.05 ±0.03	−0.22 ±0.03*	−0.29 ±0.04*	−0.38 ±0.04*	−0.10 ±0.03**	−0.15 ±0.04*	−0.26 ±0.04*
Uttarakhand	0.09 ±0.03**	0.15 ±0.03*	0.08 ±0.05	0.09 ±0.03**	0.06 ±0.03	0.01 ±0.05	−0.01 ±0.03	0.01 ±0.04	0.02 ±0.05	0.03 ±0.03	0.04 ±0.04	0.06 ±0.05
Rajasthan	0.21 ±0.02*	0.20 ±0.02*	0.12 ±0.02*	0.19 ±0.02*	0.22 ±0.02*	0.18 ±0.02*	−0.13 ±0.02*	−0.20 ±0.02*	−0.22 ±0.02*	−0.08 ±0.02*	−0.07 ±0.02*	−0.15 ±0.02*
Orissa	0.11 ±0.02*	0.16 ±0.03*	0.11 ±0.03*	0.14 ±0.02*	0.16 ±0.02*	0.09 ±0.03**	−0.07 ±0.02**	−0.15 ±0.03*	−0.13 ±0.03*	0.06 ±0.02***	0.01 ±0.02	−0.09 ±0.03**
Madhya Pradesh	0.31 ±0.01*	0.39 ±0.02*	0.22 ±0.02*	0.35 ±0.01*	0.41 ±0.02*	0.25 ±0.02*	−0.13 ±0.01*	−0.15 ±0.02*	−0.23 ±0.02*	0.01 ±0.01	0.02 ±0.02	−0.17 ±0.02*
All EAG states	0.13 ±0.01*	0.16 ±0.01*	0.05 ±0.01*	0.14 ±0.00*	0.15 ±0.01*	0.07 ±0.01*	−0.05 ±0.01*	−0.08 ±0.01*	−0.15 ±0.01*	0.03 ±0.01*	0.01 ±0.01	−0.10 ±0.00*
**NE states**												
Arunanchal Pradesh	0.08 ±0.03***	−0.003 ±0.04	−0.01 ±0.04	0.08 ±0.03**	0.12 ±0.04**	0.11 ±0.04**	0.00 ±0.03	−0.14 ±0.04**	−0.21 ±0.04*	0.08 ±0.03***	0.03 ±0.04	0.04 ±0.04
Assam	0.20 ±0.02*	0.17 ±0.03*	0.08 ±0.03**	0.18 ±0.02*	0.22 ±0.02*	0.27 ±0.03*	0.03 ±0.02	−0.09 ±0.03**	−0.31 ±0.03*	0.08 ±0.02**	0.02 ±0.03	−0.14 ±0.03*
Meghalaya	−0.03 ±0.04	0.17 ±0.04*	0.29 ±0.04*	0.04 ±0.03	0.24 ±0.03*	0.19 ±0.05*	−0.19 ±0.05*	0.03 ±0.04	0.11 ±0.05***	−0.03 ±0.04	0.08 ±0.05	−0.05 ±0.05
Sikkim	−0.15 ±0.07***	−0.21 ±0.07**	−0.22 ±0.07**	−0.06 ±0.06	−0.23 ±0.07**	−0.19 ±0.06**	0.03 ±0.07	−0.08 ±0.07	−0.29 ±0.07*	0.04 ±0.06	0.05 ±0.07	−0.08 ±0.06
Manipur	−0.09 ±0.04***	−0.08 ±0.05	−0.11 ±0.06	−0.05 ±0.03	−0.15 ±0.05**	−0.16 ±0.06**	0.21 ±0.05*	0.09 ±0.05	0.11 ±0.05***	0.11 ±0.04**	0.15 ±0.05**	−0.14 ±0.05**
Tripura	−0.25 ±0.07**	−0.36 ±0.08*	−0.15 ±0.07***	−0.32 ±0.07*	−0.20 ±0.07**	−0.04 ±0.08	−0.54 ±0.07*	−0.44 ±0.08*	−0.22 ±0.07**	−0.40 ±0.07*	−0.38 ±0.07*	−0.13 ±0.08
Mizoram	0.15 ±0.05**	0.24 ±0.05*	0.10 ±0.04***	0.05 ±0.04	−0.03 ±0.04	0.05 ±0.05	0.46 ±0.05*	0.40 ±0.05*	0.17 ±0.04*	0.24 ±0.05*	0.17 ±0.04*	0.13 ±0.05***
All NE states	0.04 ±0.01**	0.06 ±0.02*	0.04 ±0.02***	0.06 ±0.01*	0.07 ±0.02*	0.10 ±0.02*	0.02 ±0.02	−0.03 ±0.02	−0.13 ±0.02*	0.06 ±0.01*	0.04 ±0.02**	−0.06 ±0.02*

*Notes*: (i) Coefficients are average treatments effects  ± standard errors; (ii) the models were estimated separately for wealth and education tertiles, and repeated for each state and state groups, after controlling for age, rural-urban, parity and caste; (iii) *P < 0.001, **P < 0.01, *** *P* < 0.05; (iv) the estimates were from data of DLHS-1 (1995–99), DLHS-2 (2001–04) and DLHS-3 (2007–08).

In the late post-NRHM period 2011–12, we found greater equity in the uptake of institutional delivery. In the EAG states as a whole, the net increase in the uptake of institutional delivery was 49%, 42% and 18% for the lowest, middle and highest wealth tertiles, respectively (Table 3). Similarly, in the NE states, the net increase in the uptake of institutional delivery was 31%, 22% and 7% for the lowest, middle and highest wealth tertiles, respectively. Similar trends were found for the education tertiles.

**Table 3.** Effects of NRHM in the uptake of institutional delivery and ante-natal care among wealth and education tertiles in the late post-NRHM period 2011−12, in high-focus empowered action group (EAG) and north eastern (NE) Indian states

	Institutional delivery						Ante-natal care					
	Wealth tertiles			Education tertiles			Wealth tertiles			Education tertiles		
	Lowest tertile (1)	Middle tertile (2)	Highest tertile (3)	Lowest tertile (4)	Middle tertile (5)	Highest tertile (6)	Lowest tertile (7)	Middle tertile (8)	Highest tertile (9)	Lowest tertile (10)	Middle tertile (11)	Highest tertile (12)
**EAGstates**												
Jharkhand	0.24 ±0.02*	0.27 ±0.02*	0.20 ±0.03*	0.22 ±0.01*	0.23 ±0.02*	0.18 ±0.02*	0.38 ±0.02*	0.47 ±0.03*	0.30 ±0.03*	0.39 ±0.02*	0.33 ±0.02*	0.19 ±0.03*
Uttar Pradesh	0.45 ±0.01*	0.39 ±0.01*	0.18 ±0.01*	0.41 ±0.01*	0.39 ±0.01*	0.28 ±0.01*	0.22 ±0.01*	0.18 ±0.01*	0.12 ±0.01*	0.26 ±0.01*	0.28 ±0.01*	0.28 ±0.01*
Bihar	0.46 ±0.01*	0.45 ±0.01*	0.19 ±0.02*	0.46 ±0.01*	0.31 ±0.01*	0.06 ±0.02*	0.31 ±0.01*	0.34 ±0.01*	0.08 ±0.02*	0.39 ±0.01*	0.27 ±0.01*	−0.07 ±0.02*
Chhattisgarh	0.26 ±0.02*	0.29 ±0.02*	−0.06 ±0.03***	0.29 ±0.02*	0.27 ±0.02*	−0.08 ±0.03**	0.10 ±0.03*	−0.12 ±0.03*	−0.28 ±0.03*	0.14 ±0.03*	0.04 ±0.03	−0.17 ±0.03*
Uttarakhand	0.38 ±0.03*	0.42 ±0.03*	0.31 ±0.04*	0.30 ±0.02*	0.35 ±0.03*	0.23 ±0.05*	0.32 ±0.03*	0.25 ±0.03*	0.20 ±0.04*	0.31 ±0.03*	0.32 ±0.03*	0.22 ±0.05*
Rajasthan	0.53 ±0.01*	0.48 ±0.02*	0.29 ±0.02*	0.53 ±0.01*	0.46 ±0.02*	0.34 ±0.02*	0.26 ±0.01*	0.17 ±0.02*	0.05 ±0.02**	0.29 ±0.01*	0.29 ±0.02*	0.13 ±0.02*
Orissa	0.51 ±0.02*	0.35 ±0.02*	0.09 ±0.02*	0.49 ±0.01*	0.38 ±0.02*	0.04 ±0.02	0.34 ±0.02*	0.11 ±0.03*	−0.05 ±0.02	0.44 ±0.02*	0.28 ±0.02*	−0.02 ±0.02
Madhya Pradesh	0.65 ±0.01*	0.57 ±0.01*	0.26 ±0.02*	0.65 ±0.01*	0.57 ±0.01*	0.27 ±0.02*	0.42 ±0.01*	0.29 ±0.02*	0.00 ±0.02	0.49 ±0.01*	0.43 ±0.02*	0.06 ±0.02*
All EAG states	0.49 ±0.00*	0.42 ±0.01*	0.18 ±0.01*	0.44 ±0.00*	0.40 ±0.01*	0.19 ±0.01*	0.31 ±0.01*	0.22 ±0.01*	0.07 ±0.01*	0.34 ±0.00*	0.33 ±0.01*	0.09 ±0.01*
**NE states**												
Arunanchal Pradesh	0.06 ±0.03**	0.10 ±0.04*	0.00 ±0.04	0.09 ±0.03*	0.15 ±0.03*	0.13 ±0.04*	−0.05 ±0.03	−0.09 ±0.04**	−0.14 ±0.04*	0.05 ±0.03	0.06 ±0.03	0.08 ±0.04***
Assam	0.55 ±0.01*	0.43 ±0.02*	0.19 ±0.03*	0.49 ±0.01*	0.47 ±0.02*	0.42 ±0.03*	0.40 ±0.02*	0.13 ±0.03*	−0.19 ±0.03*	0.43 ±0.02*	0.43 ±0.02*	0.01 ±0.03
Meghalaya	0.15 ±0.04*	0.42 ±0.04*	0.45 ±0.05*	0.21 ±0.03*	0.41 ±0.04*	0.33 ±0.05*	0.04 ±0.05	0.23 ±0.05*	0.25 ±0.05*	0.17 ±0.04*	0.19 ±0.05*	0.13 ±0.05*
Sikkim	0.24 ±0.06*	0.13 ±0.07	−0.16 ±0.07**	0.24 ±0.06*	0.06 ±0.07	−0.09 ±0.06	0.15 ±0.06***	−0.03 ±0.07	−0.23 ±0.06*	0.12 ±0.06***	0.08 ±0.07	−0.09 ±0.06
Manipur	0.05 ±0.05	0.12 ±0.05**	−0.09 ±0.05	0.08 ±0.03**	−0.07 ±0.05	−0.14 ±0.06**	0.22 ±0.05*	0.12 ±0.05***	−0.01 ±0.05	0.08 ±0.04***	0.14 ±0.05**	−0.26 ±0.05*
Tripura	0.09 ±0.07	−0.10 ±0.08	0.03 ±0.07	−0.09 ±0.07	−0.01 ±0.07	0.06 ±0.07	−0.20 ±0.07**	−0.22 ±0.08**	−0.15 ±0.07***	−0.22 ±0.07*	−0.17 ±0.07**	−0.14 ±0.07
Mizoram	0.28 ±0.05*	0.41 ±0.05*	0.17 ±0.04*	0.21 ±0.04*	0.09 ±0.04**	0.09 ±0.04***	0.38 ±0.05*	0.40 ±0.05*	0.19 ±0.04*	0.24 ±0.04*	0.08 ±0.04	0.10 ±0.05***
All NE states	0.42 ±0.01*	0.31 ±0.02*	0.15 ±0.02*	0.36 ±0.01*	0.34 ±0.01	0.29 ±0.02*	0.33 ±0.01*	0.12 ±0.02*	−0.07 ±0.02*	0.34 ±0.01*	0.20 ±0.02*	0.04 ±0.02**

*Notes*: (i) Coefficients are average treatments effects  ± standard errors; (ii) the models were estimated separately for wealth and education tertiles, and repeated for each state and state groups, after controlling for age, rural-urban, parity and caste; (iii) *P < 0.001, **P < 0.01, ***P < 0.05; (iv) the estimates were from data of DLHS-1 (1995–99), DLHS-2 (2001–04) and DLHS-4/AHS (2011–12).

On the contrary, the observed probability in the uptake of ANC was lower than the predicted probability in both EAG and NE states for each socioeconomic tertile in the early post-NRHM period 2007–08 (Supplementary Figure S1 and S2). Nevertheless, negative effects in the uptake of ANC were found for each socioeconomic tertile in the early post-NRHM period 2007–08 (Table 2). In the EAG states as a whole, the uptake of ANC for the lowest, middle and highest wealth tertiles decreased by 5.3% (*β*=−0.053; *P* < 0.001), 8.0% (*β*=−0.080; *P* < 0.001) and 15.1% (*β*=−0.151; *P* < 0.001), respectively. In the NE states, there was no significant effects for the uptake of ANC for the lowest and middle wealth tertiles, but negative effects for the highest wealth tertiles (*β*=−0.131; *P* < 0.001). However, in the late post-NRHM period 2011–12, there was considerable improvement in the uptake of ANC, particularly for the lowest socioeconomic tertiles (Table 3).

### Interstate variations on the impacts of NRHM

Considerable inter-state variations were found on the population level effects of NRHM in the uptake of both institutional delivery and ANC. Of the eight EAG states, in the early post-NRHM period 2007–08, no impact of NRHM on the uptake of institutional delivery was found in the states of Jharkhand but positive impact was found on the rest of the EAG states (Table 2). Of the seven NE states, negative effect of NRHM in the uptake of institutional delivery was found among the low wealth tertiles in the states of Sikkim, Manipur and Tripura, and no effect in Meghalaya. However, in the late post-NRHM period 2011–12, the lowest and middle wealth and education tertiles of the most states, except Manipur and Tripura, had positive impact in the uptake in institutional delivery. In the uptake of ANC in the early post-NRHM period 2007–08, positive effect of NRHM was found particularly among the low wealth tertile in the EAG states of Jharkhand and Bihar, and negative effects in the remaining six EAG states. In the NE states, among the low wealth tertile, no impact in the uptake of ANC in Arunanchal Pradesh, Assam and Sikkim, however, positive impact in Manipur and Mizoram, and negative impact in Meghalaya and Tripura, was found. In the late post-NRHM period 2011–12, positive impacts on the uptake of ANC were found among low and middle socioeconomic tertiles in most of the states, except Arunachal Pradesh, Meghalaya and Tripura.

On one hand, we also found that the effects of JSY coverage and NRHM in the uptake of institutional delivery and ANC showed a similar pattern. For instance, in the early post-NRHM period 2007–08, in Jharkhand where only 3.3% of the eligible women had received JSY cash-transfer, there was no population level impact of NRHM in the uptake of institutional delivery but positive impacts in the uptake of ANC. But, in the late post-NRHM period 2011–12, 28% of the eligible women had received JSY cash-transfer, and this was associated with positive impacts in the uptake of institutional delivery as well. On the other hand, the three EAG states with higher levels of JSY coverage in 2007–08, namely, Rajasthan (32.5%), Orissa (38.9%) and Madhya Pradesh (42.8%) had positive population level effects in the uptake of institutional delivery.

### Variation in impacts between wealth and education groups

We found almost similar pattern on the RII and the effects between wealth and education measures of socioeconomic position. For example, in the EAG states in the early post-NRHM period 2007–08, the effects of NRHM in the uptake of institutional delivery for the lowest (*β* = 0.133; *P* < 0.001), middle (*β* = 0.157; *P* < 0.001) and highest wealth tertiles (*β* = 0.053; *P* < 0.001) are similar to the lowest education (*β* = 0.14; *P* < 0.001), middle education (*β* = 0.15; *P* < 0.001) and highest education tertiles (*β* = 0.07; *P* < 0.001). However, there were few instances of inconsistent pattern of our results in some states between wealth and education tertiles.

### JSY coverage and inequity over time

Only 17.9% of the eligible women in the EAG states (ranging from 3% in Jharkhand to 43% in Madhya Pradesh) and 18.5% of the eligible women in the NE states (ranging from 3% in Meghalaya to 30% in Mizoram) received financial incentives from the JSY in the early post-NRHM period 2007–08 (Supplementary Table S1). There was an increase in the percentage of eligible women receiving financial incentives in the late post-NRHM period 2011–12, being 48.6% in the EAG (ranging from 28% in Jharkhand to 76.6% in Madhya Pradesh) and 56.3% in the NE states (ranging from 12.7% in Meghalaya to 56.7% in Assam). Furthermore, there was inequity in the receipt of JSY, that is, people from higher socioeconomic groups benefited more than the people from lower socioeconomic groups in the early post-NRHM period 2007–08, and this pattern had reversed or attenuated in the late post-NRHM period 2011–12. The wealth-related RII in receipt of JSY incentives declined from 1.3 (95% CI: 1.2; 1.4) in 2007–08 to 0.95 (95% CI: 0.94; 0.97) in 2011–12 in the EAG states and from 3.6 (95% CI: 2.8; 4.7) to 1.14 (95% CI: 1.10; 1.19) in the NE states.

### Robustness

Besides using the RII for measuring the inequity, we used both the slope index of inequality and concentration index, another widely used measures of inequity that take the whole socioeconomic distribution of population into account, and we found that a similar pattern of inequity in the uptake in the pre- and post-NRHM periods. The DiD estimates were re-estimated for different wealth and education quintiles and quartiles, and found the effects similar to that of the tertiles.

We estimated changes in the uptake of skilled birth-attendance at home in the pre- and post-NRHM periods. We found that the percentage of eligible women who used skilled birth-attendance at home increased between the pre-NRHM Period 1 (1995–99) and the pre-NRHM Period 2 (2000–04), but decreased in the post-NRHM period (2007–08). In the EAG states as a whole, the percentage of women who used skilled birth-attendance at home increased from 13% to 14% in the pre-NRHM periods and then decreased to 5% in the early post-NRHM period 2007–08, and further increased to 8% in the late post-NRHM period 2011–12. The corresponding estimates for the NE states are 10–12% in the pre-NRHM periods to 5% in the early post-NRHM period 2007–08 to 5% in the late post-NRHM period 2011–12. We further estimated the skilled birth-attendance consisting of both institutional delivery and skilled birth-attendance at home, and found that there was only moderate increase in the early post-NRHM period 2007–08 but considerable increase in the late post-NRHM period 2011–12. That is, over the time periods of 1995–99, 2000–04, 2007–08 and 2011–12, the skilled birth-attendance had increased from 31% to 39% to 43% to 73% in the EAG states and 39% to 47% to 48% to 72% in the NE states.

We also tested the quality of ANC by examining whether there were changes in the uptake of the Iron Folic Acid (IFA) supplementation and the Tetanus Toxoid (TT) injections. We found, on one hand, in 1995–99, 2000–04, 2007–08 and 2011–12, there was improvement in the use of IFA from 33% to 49% to 79% to 88% in the EAG states, respectively. On the other hand, there was decrease in the uptake of TT injection in EAG states in 2007–08 (from 65% to 74% to 65%) but again increased to 97% in 2011–12. In the NE states, there was an increase in the uptake of TT injection over time (from 61% to 64% to 71% to 96%).

## Discussion

In this study, we assessed the population-level impact of NRHM on socioeconomic inequities in the uptake of two major components of maternal health services, namely institutional delivery and ANC. We found evidence supporting our hypotheses that the effect of NRHM in the uptake of institutional delivery among pregnant women from lower socioeconomic backgrounds was greater than the effect on women from higher socioeconomic backgrounds. The inequities in institutional delivery and ANC were already declining between the pre-NRHM Period 1 (1995–99) and the pre-NRHM Period 2 (2000–04), but declined at steeper rates in the post-NRHM periods. The effects were stronger for institutional delivery. Our findings also support our hypothesis that the effects of NRHM on the increase in the uptake and the decline in socioeconomic inequities were greater for institutional delivery than ANC in the early post-NRHM period 2007–08. This pattern was more evident in those states with higher proportion of eligible women enrolled under JSY cash-transfer. One of the reasons can be that the conditional cash-transfer linked to institutional delivery might have attracted huge attention to the promotion of institutional delivery, and thus neglecting other components of maternal and child healthcare such as ANC and child immunisation. This finding highlights the need within NRHM to link antenatal services with institutional delivery to achieve a continuity of maternal health services (Lahariya 2009). Equity and uptake of institutional delivery and ANC improved in most states in late post-NRHM period 2011–12, the period when the targeted outreach of the most components of the NRHM was reached.

Our study also found considerable inter-state variations in the impacts of the NRHM and the proportion of eligible women that received cash transfers. To address these inter-state variations, the central government in collaboration with the low performing state governments needs to implement appropriate policy measures, including of ensuring the availability of functional and trained ASHAs in each village, increased cash-transfer amount for the pregnant women, higher incentives for the ASHAs and good quality health services at the public health facilities. Furthermore, conducting state-wise in-depth evaluation studies, using both quantitative and qualitative methods, for identifying the underlying reasons for the inter-state variations would be useful for formulating effective policy guidelines.

Over the past few years, several low- and middle-income countries including Mexico, Columbia, Nicaragua, Honduras and Brazil have introduced public health and social security programs with cash-transfer to increase the use of health services by poor people (Attanasio *et al.* 2005). Studies have found increase in the use of health services and health status (Gertler 2000; Morris 2004; Rivera 2004; Maluccio and Flores 2005; Lagarde *et al.* 2009). For example, ‘Familias en Accion’ of Columbia improved the nutritional status and reduced the morbidity of young children (Attanasio *et al.* 2005). The ‘Bolsa Alimentação’ of Brazil also improved health care use by children (Shei 2014). A recent study found that the combined effects of Brazil’s massive expansion of primary health care via the ‘Family Health Program’ and the cash-transfer of ‘Bolsa Alimentação’ successfully reduced post-neonatal infant mortality (Guanais 2013).

Several studies have assessed the impact of India’s JSY (Lim *et al.* 2010; Modugu 2013; Carvalho 2014; Randive 2014), and we have added to this evidence base by assessing the overall impact of NRHM at the population level. Our impact assessment included the larger components of the program including the JSY payments, the large scale public healthcare investment followed after 2005, and the deployment of grassroots level health workers (i.e. ASHA), and several healthcare committees at various levels including those with community involvement for enhancing the uptake of primary healthcare. Further, the nationally representative household level data covering major Indian states, which were collected independently of the NRHM, enabled us to perform a comparative assessment of impacts of NRHM between the states. In addition, to ensure more robust impact assessment we used two pre-NRHM period data, as suggested by the literature of impact assessment in other contexts (Wagstaff 2009, 2010). The use of two pre-NRHM and two post-NRHM data points had helped us to apply a quasi-natural experiment framework to control for potential confounders and secular trends. The NRHM was changed to the National Health Mission (NHM) in 2013, which is now made up of NRHM and the National Urban Health Mission. The latter element was launched to meet the primary health care needs of the urban population with a focus on the urban poor. The use of the latest available data set of DLH-4 and AHS had allowed us to assess the impact of a more comprehensive form of this public health program after its almost full implementation and widest outreach.

Our study has several limitations. First, the NRHM is a targeted public health program intending to enhance the uptake of primary healthcare especially of lower socioeconomic groups, but is characterised as a population level intervention where all segments of the population can benefit. This makes it difficult to define the treatment and control group in strict sense as per standard impact evaluation methodology. Instead, we opted to define lower socioeconomic groups as target groups and the higher socioeconomic as the non-target groups. Second, we estimated the effects on the assumption of a linear increase in the uptake of maternal health services in the post-NRHM periods based on the trends of the pre-NRHM Periods 1 and 2. This assumption may not hold true in the real world setting, and increases in the uptake over the period of time are likely to occur at diminishing rates as it becomes increasingly difficult to reach the most vulnerable population groups. However, since we used both institutional delivery and ANC as maternal health services outcomes for comparative assessment, we assume that these possible external factors would in general affect both outcomes in a similar way.

The study findings have important implications for health policy in India and other developing countries, particularly in the present context of growing debate in India and other developing countries on various strategies of developing the health system and achieving universal coverage, which is centred on two paths: strengthening the supply side of the public healthcare system along with targeted interventions and strengthening demand-side financing by promoting health insurance programmes with larger roles for private healthcare providers (World Health Organization 2010). The report of the High Level Expert Group (HLEG) established by the Indian Planning Commission recommended for strengthening the public healthcare system instead of relying on the health insurance option (Planning Commission 2011). However, recently, concerns have been raised about India’s healthcare policy shifts, including the proposal to reduce government funds for NRHM, and to rely more on the private health sector in primary healthcare (Staff Reporter 2014; Reeves *et al.* 2015). Our findings suggest that strengthening public healthcare infrastructure, using public health intervention programs with focus on the weaker sections of the society and increased resource allocation, will enhance the uptake of maternal healthcare, improve health outcomes and contribute to the achievement of the health-related Millennium Development Goals. Giving due considerations to the effective design and implementation of the public health programs by linking various components of maternal and child healthcare will improve universal access to comprehensive healthcare. Previous studies have argued that socially disadvantaged individuals with less education and living in places with poor health facilities fail to perceive the need for accessing healthcare (Gulliford *et al.* 2002; Sen 2002; Vellakkal *et al.* 2013). Our results indicate that over-coming financial and other structural barriers through programs focussing lower socioeconomic groups, rather than psychological perceptions of poor people, are likely to promote uptake and reduce inequities in uptake of maternal and child healthcare.

## Supplementary Data 

Supplementary data are available at *HEAPOL* online

## Supplementary Material

Supplementary Data
