# Supplementary material for: Has India’s national rural health mission reduced inequities in maternal health services? A pre-post repeated cross-sectional study
Source: Health Policy Plan. 2016 Aug 10;32(1):79–90. doi: 10.1093/heapol/czw100 (PMC5886191; doi:10.1093/heapol/czw100)
Supplement: Supplementary Data [file czw100_supp.docx]

Supplementary Figure S1. Observed and predicted probability (95% CIs) in the uptake of institutional delivery and ante-natal care among wealth tertiles, in high-focus empowered action group (EAG) and north eastern (NE) Indian states

|  |  |
| --- | --- |
|  |  |

Notes: i) Predicted probability in the uptake of institutional delivery and ante-natal care in 2007-08 and 2011-12 was based on the trends in the uptake between 1995-99 and 2000-04; ii) Estimates were adjusted for age, rural-urban, caste, parity, state level fixed effects; iii) Error bars denote 95% confidence intervals

Supplementary Figure S2. Observed and predicted probability (95% CIs) in the uptake of institutional delivery and ante-natal care among education tertiles, in high-focus empowered action group (EAG) and north eastern (NE) Indian states

|  |  |
| --- | --- |
|  |  |

Notes: i) Predicted probability of the uptake of institutional delivery and ante-natal care in 2007-08 and 2011-12 was based on the trends of the uptake between 1995-99 and 2000-04; ii) Estimates were adjusted for age, rural-urban, caste, parity and state level fixed effects; iii) Error bars denote 95% confidence intervals
